# Examining the Evidence Regarding Smoking and Patient Outcomes for Isolated Meniscus Pathology: A Comprehensive Systematic Review and Meta-Analysis

**DOI:** 10.3390/life14050584

**Published:** 2024-04-30

**Authors:** Omkar Anaspure, Shiv Patel, Anthony N. Baumann, Albert T. Anastasio, Kempland C. Walley, John D. Kelly, Brian C. Lau

**Affiliations:** 1 Perelman School of Medicine, University of Pennsylvania, Philadelphia, PA 19104, USA; shiv.patel@pennmedicine.upenn.edu; 2 College of Medicine, Northeast Ohio Medical University, Rootstown, OH 44272, USA; abaumann@neomed.edu; 3 Department of Orthopedic Surgery, Duke University, Durham, NC 27710, USA; albert.anastasio@gmail.com (A.T.A.); brian.lau@duke.edu (B.C.L.); 4 Department of Orthopaedic Surgery, University of Michigan Health System, Ann Arbor, MI 48109, USA; kcwalley@med.umich.edu; 5 Department of Orthopaedic Surgery, University of Pennsylvania, Philadelphia, PA 19104, USA; john.kelly@pennmedicine.upenn.edu

**Keywords:** meniscus, meniscectomy, smoking, tobacco, meniscus tear, meniscus repair

## Abstract

Smoking is a well-known cause of impairment in wound healing and postoperative outcomes; however, its effects on treating meniscus issues remain unclear. This study assesses the relationship between smoking and meniscus treatment outcomes. PubMed, Scopus, Cochrane, and CINAHL were searched from inception to 24 December 2023. Inclusion criteria encompassed studies examining smoking’s impact on patient outcomes regarding meniscus pathology. A secondary PubMed search targeted randomized controlled trials (RCTs) in the top ten orthopedic journals focusing on meniscus pathology and smoking as a demographic variable. Meta-analysis of six studies (*n* = 528) assessed meniscus failure rate based on smoking status. Eighteen observational studies (*n* = 8353 patients; 53.25% male; mean age: 51.35 ± 11.53 years; follow-up: 184.11 ± 117.34 months) were analyzed, covering meniscus repair, meniscectomy, allograft transplant, conservative care, and arthroscopy. Results showed four studies (36.36%) linked smoking with worse meniscus repair outcomes, while seven studies (63.64%) did not find significant associations. Meta-analysis from six studies showed no significant impact of smoking on repair failure (*p* = 0.118). Regarding meniscectomy, one study (33.33%) identified a significant association with smoking, but two did not. Only one (3.8%) of the RCTs in leading orthopedic journals included smoking as a factor. The evidence on smoking’s effect on meniscus treatment is mixed, necessitating further investigation.

## 1. Introduction

The menisci in the knee joint play crucial roles supporting joint function, such as acting as shock absorbers, which reduces pressure on the joint and prevents damage [1,2,3,4,5,6]. The meniscus aids with knee stability, allows smooth movement by enhancing synovial fluid distribution, and limits excessive motion, thereby reducing the risk of injury. They are also significant for proprioception, aiding in movement coordination [5,6]. Thus, healthy menisci are essential for mobility, stability, and the knee’s shock-absorbing capabilities. Meniscus injuries are common and can lead to long-term complications like pain and osteoarthritis, resulting in a significant personal and economic burden [7,8,9,10]. The incidence of meniscal injuries is notable in both athletic and general populations, often requiring surgical intervention [1,7,10,11]. In the United States, the incidence rate for meniscal tears is 61 per 100,000 in the general population, with active-duty military personnel experiencing a higher rate of 8.7 per 1000 [12]. As studies seek to identify risk factors for poor outcomes in meniscus pathology, the influence of smoking on tissue healing and repair has emerged as a suggested risk factor [13,14,15,16]. Smoking is known to adversely affect wound healing and increase surgical complication risks, particularly in orthopedic conditions [17,18,19,20]. Smoking’s adverse effects involve impaired blood flow, reduced oxygenation, and altered inflammatory responses, all critical for tissue healing [20,21,22,23]. Impaired blood flow is especially relevant in the meniscus, which has limited baseline blood supply, especially in its inner portion [2,24,25]. This is concerning due to the global prevalence of smoking, the meniscus’s low blood supply, and its potential to complicate postoperative recovery in orthopedic patients with meniscal injuries [19].

Despite the well-accepted impact of smoking on various orthopedic conditions, its influence on patient outcomes for meniscal pathology remains unclear [18,26,27,28]. Surprisingly, there is a lack of research specifically focusing on the relationship between smoking and meniscus injury outcomes [29]. This gap is significant, given the potential for smoking to exacerbate postoperative complications, delay return to function, and affect the success of meniscus repair or reconstruction surgeries. No systematic review has addressed this issue, limiting consensus and potentially impacting patient risk assessment.

Therefore, this study aims to examine the association between smoking and patient outcomes for meniscal pathology while assessing the current literature to improve patient education and risk stratification. Understanding this relationship is essential for developing targeted strategies to enhance patient outcomes, inform preoperative counseling, and guide postoperative care in smokers with meniscus injuries.

## 2. Materials and Methods

### 2.1. Information Sources and Search Strategy

This systematic review examines the impact of smoking on meniscus tears and postoperative retears or failures for patients with varying meniscus injury. PubMed, SCOPUS, COCHRANE, and MEDLINE databases were searched from inception until 24 December 2023. Search terms used in each database were as follows: (“smoking” OR “smoke” OR “smoker” OR “nicotine” OR “tobacco” OR “cigarette” OR “cigar” OR (cigarette smoking[MeSH Terms]) OR (smoking, cigarette[MeSH Terms])) AND (“meniscus” OR “meniscus tear” OR “meniscus repair” OR “meniscectomy”). This study was performed under the guidelines of the most recent Preferred Reporting Items for Systematic Reviews and Meta-Analyses (PRIMSA) for proper data reporting.

### 2.2. Inclusion and Exclusion Criteria

Inclusion criteria were randomized controlled trials (RCTs) or observational studies that included patients with meniscus tears as an isolated injury, patients undergoing surgical intervention for a meniscus tear, and smoking as a study demographic factor. Exclusion criteria were studies that had non-isolated meniscus tears, studies that provided interventions for the meniscus pathology secondary to another injury, or studies that did not include smoking as a study factor.

### 2.3. Article Screening Process

This systematic review used Rayyan to assist with article sorting. After articles were downloaded into Rayyan, duplicates were removed and then articles were sorted by title and abstract according to the inclusion and exclusion criteria. Studies were then screened by full text for final study inclusion.

### 2.4. Data Extraction

Data were extracted by multiple authors during this study. Data extracted included study type, type of surgery, total patients, sex, patient age, follow-up time, significant findings regarding smoking and tears, and number of postoperative failures.

### 2.5. Article Quality Grading

All observational studies included in this systematic review were classified as either “comparative” or “non-comparative” to appropriately grade their quality using the Methodological Index for Non-Randomized Studies (MINORS) scale [30]. Comparative studies were graded out of 24 points and non-comparative studies were graded out of 16 points. For comparative studies, there are 8 items on the scale, and for non-comparative studies, there are 12 items on the scale with each item being rated from 0 to 2 points. All articles were considered to be “high-quality”, “moderate-quality”, or “low-quality” based on their scoring and comparative/non-comparative nature. For comparative studies, high-quality articles scored 24 points, moderate-quality articles scored 15–23 points, and low-quality articles scored less than 15 points [31]. For non-comparative studies, high-quality articles scored 16 points, moderate-quality articles scored 10–15 points, and low-quality articles scored less than 10 points [31].

### 2.6. Additional Literature Search on Randomized Controlled Trials

To further assess the literature on this topic, a secondary search was completed to evaluate the frequency of smoking reporting as a patient demographic in RCTs in the top ten orthopedic journals by impact factor by searching the Observatory of International Research (OOIR) website (Table 1). The complete search query for this search was as follows: ((“J Physiother” [Journal]) OR (“Osteoarthritis Cartilage” [Journal]) OR (“Am J Sports Med” [Journal]) OR (“J Bone Joint Surg Am” [Journal]) OR (“J Orthop Sports Phys Ther” [Journal]) OR (“Knee Surgery, Sports Traumatology, Arthroscopy: Official Journal of the ESSKA” [Journal]) OR (“The Journal of Arthroplasty” [Journal]) OR (“Bone Joint J” [Journal]) OR (“Clinical Orthopaedics and Related Research” [Journal]) OR (“Acta Orthopaedica” [Journal])) AND (“Meniscus” OR “Meniscus Tear” OR “Meniscus Injury” OR “Menisci, Tibial/Surgery” [MAJR] OR “Tibial Meniscus Injuries” [MeSH] OR “Menisci, Tibial/Pathology” [MeSH]) OR “Menisci, Tibial/Surgery” [MeSH]) [13,14,15,16,32,33,34,35,36,37,38,39,40,41,42,43,44]. Each article was then searched for any mention of smoking as a demographic variable.

### 2.7. Statistical Analysis

The Statistical Package for the Social Sciences (SPSS) version 29.0 (Armonk, NY, USA: IBM Corp.) was used for this study. Descriptive statistics such as frequency-weighted means were used to describe the data. Due to study heterogeneity, a narrative approach to systematic review was used for results. A random-effects binary outcomes meta-analysis was performed using odds ratio (OR) and 95% confidence intervals (CIs) for effect size. OR was used due to the nature and study design of the included studies. Zero cases were adjusted to 0.5 for binary outcomes as elsewhere in the literature. A forest plot was generated to depict the relationship between variables.

## 3. Results

### 3.1. Initial Search Results

**Figure 1:**
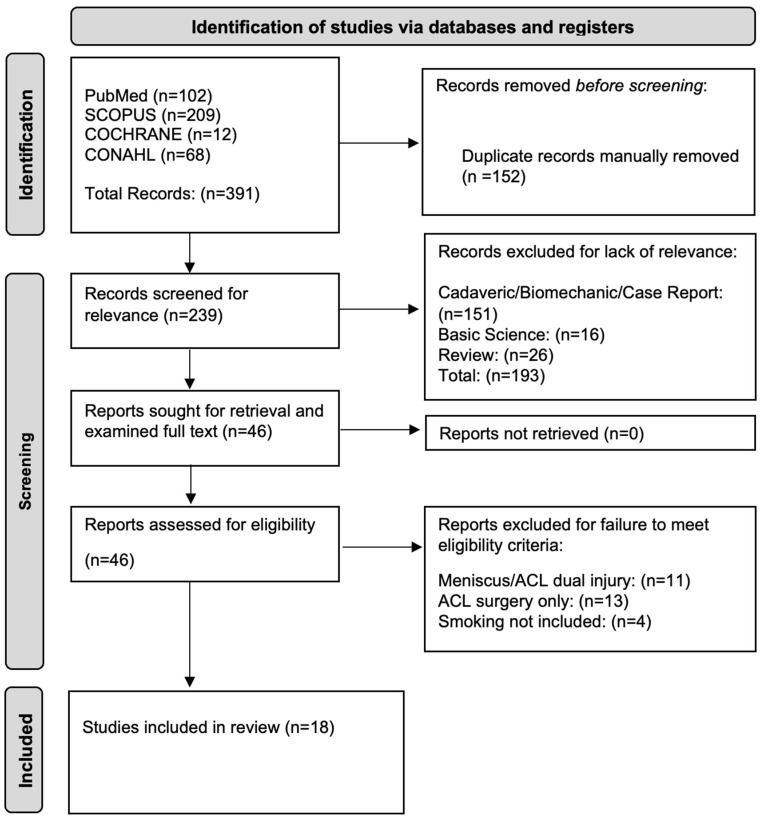
The Preferred Reporting Items for Systematic Reviews and Meta-Analyses (PRISMA) diagram outlining the entire search progress, from initial search in four databases to final article inclusion.

A total of 18 articles met the inclusion criteria from the 391 articles retrieved during the primary search for this systematic review (Figure 1).

### 3.2. Article Quality Results

Eighteen articles were observational studies (fifteen retrospective, three prospective) graded according to the MINORS scale (Table 2) [30]. The mean MINORS score for all included articles was 13.1 ± 3.2 points, with eleven articles being comparative and seven articles being non-comparative studies. The mean MINORS score for non-comparative studies (*n* = 7 articles) was 10.0 ± 1.2 points out of a possible 16.0 points, and the mean MINORS score for comparative studies (*n* = 11 articles) was 15.1 ± 2.3 points out of a possible 24.0 points. Eleven articles were designated as “moderate-quality” based on the MINORS scale, seven articles were designated as “low-quality”, and no articles were designated as “high-quality”.

### 3.3. Patient Demographics

Total patients (*n* = 8353; 53.25% male) had a frequency-weighted mean age of 51.35 ± 11.53 (*n* = 8301; 100.0% of patients) with a frequency-weighted mean follow-up time of 184.11 ± 117.34 (*n* = 7663; 100.0% of patients) (Table 3). From our included articles (*n* = 18), 1261 patients underwent meniscus repair, 1427 patients underwent meniscectomy, and 262 patients underwent meniscus allograft transplant, and 638 patients underwent arthroscopic evaluation for meniscus pathology. All included patients (*n* = 8301) had meniscus pathology for which 3588 patients (43.22% of patients) underwent surgical intervention, and 4713 patients (56.78% of patients) underwent conservative treatment.

### 3.4. The Impact of Smoking on Outcomes for Meniscus Repair

**Figure 2:**
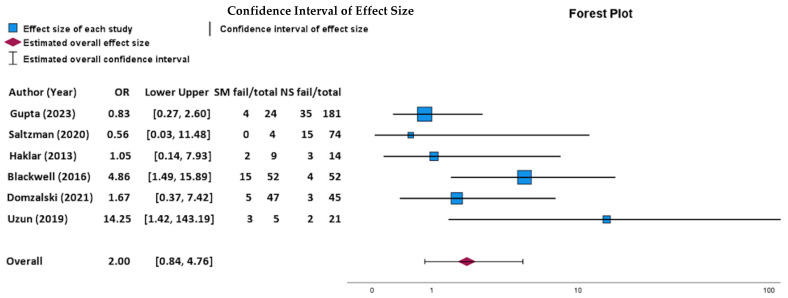
Random-effects model forest plot demonstrating relationship between meniscus failure and smoking status. Effect measures used were ORs and CIs. Abbreviations: SM, smokers; NS, nonsmokers; fail; failure; OR, odds ratio; CI, confidence interval. Heterogeneity: Tau-squared = 0.44, H-squared = 1.64, I-squared = 0.39. Axis is shown using log scale [14,15,32,33,36,43].

For smoking and meniscus repair, four studies (36.36%) found a significant association and seven studies did not (63.64%) with six studies providing number of failures stratified by smoking status (Table 4). When examining only the six studies that reported the number of failures stratified by smoking status, there was no statistically significant association between failure after meniscus repair and smoking status (*p* = 0.118; OR: 2.00; 95% CI: 0.84, 4.76; Figure 2). Pelletier et al. (2023) [13] found that smoking was a failure risk and significantly associated with bucket-handle tears in 122 patients who smoked, had bucket-handle tears, and received a medial meniscus suture repair (OR = 5.76, CI = 1.81–18.35, *p* = 0.003). Patients experienced an overall failure rate of 42%. Blackwell et al. (2016) [14] found that meniscus repair failure was 3.8 times higher for smokers vs. nonsmokers (*p* = 0.0076). To be specific, 15 smoking patients out of 52 total smoking patients (28.85%) experienced meniscus repair failure compared to a 7.69% failure rate in nonsmokers (four failures out of 52 nonsmoking patients). Domzalski et al. (2021) [15] showed that function was significantly better in nonsmokers with an average Knee Injury and Osteoarthritis Outcome Score (KOOS) score of 80.2 vs. 67.4 in the smoker group in a study of 92 patients (*p* < 0.0001). A delayed time of return to daily activities in the patient population that smoked (5.4 vs. 4.2 months) compared to nonsmokers was seen. The failure rate was 10.63% for smokers compared to 6.67% in nonsmokers. In contrast, Gupta et al. (2023) [32] found that smoking has no effect on the failure of meniscal repairs in a study of 205 patients with bucket-handle meniscus tears who underwent a meniscus repair (*p* = 0.821). This study also reported an overall 19% rate of failure postoperatively, with outside-in repair technique (33.33%) having a higher rate of failure than an all-inside technique alone (14.17%). The failure rate was 16.7% for smokers compared to 19.3% in nonsmokers. Similarly, Saltzman et al. (2020) [33] found smoking not significantly associated with failure for 75 patients undergoing meniscus repair for bucket-handle meniscus tears (*p* = 0.999), with a postoperative repair failure rate of 20%. The failure rate was 0% for smokers compared to 20.3% in nonsmokers. Buyukkuscu et al. (2019) [34] also found in a study of 33 patients with longitudinal medial meniscus tears that smoking and meniscus repair was not significantly associated with clinical and functional improvement postoperatively (*p* = 0.458). Both Laurendon et al. (2017) [39] and Haklar et al. (2013) [36] found that meniscal repair and smoking was not significantly associated with failure (*p* = 0.375, *p* > 0.05, respectively). Laurendon et al. (2017) [39] had a study of 87 patients with meniscal tear who underwent a standard arthroscopic repair procedure, with patients having a 14.90% rate of repair failure postoperatively. Haklar et al. (2013) [36] chose to perform inside-out single or double vertical sutures meniscus repair for their 112 patients and found no association between repair approach and failure related to smoking (*p* > 0.05). The failure rate was 22.2% for smokers compared to 21.4% in nonsmokers. Similarly, the Cleveland Clinic et al. (2020) [42] showed that smoking had no effect on healing in isolated meniscal tears for 145 patients with meniscus tears who underwent meniscus surgery (*p* > 0.05); these patients, however, did experience a 7.5% postoperative failure which led to reoperation. Uzun et al. evaluated 43 full-thickness lateral meniscal repairs (22 (51.2%) vertical longitudinal tears and 21 (48.8%) for bucket-handle tears). Of these, 26 patients presented with isolated meniscus injury. Uzun et al. [43] identified smoking a risk factor for repair failure for both vertical–longitudinal and bucket-handle repairs (*p* < 0.05). The failure rate was 60% for smokers compared to 9.52% in nonsmokers.

### 3.5. The Impact of Smoking on Outcomes after Meniscectomy

For meniscectomy and smoking, one article (33.33%) found no significant association between smoking and outcomes after meniscectomy whereas two articles (66.67%) found a significant association. Santana et al. (2022) [40] found no significant difference in smoking status between patients who had no tear and patients who had a tear with or without surgery in a study of 432 patients who underwent arthroscopic partial meniscectomy (*p* = 0.18). Additionally, they found that there was no significant difference in joint space width between baseline and 72 months after surgery between the nonsurgical and no-tear groups (*p* = 0.12). Jones et al. (2020) [35] found current smoking to be a modifiable risk factor significantly associated with reduced improvement in KOOS Pain and KOOS Quality of Life (QOL) scores, ultimately predicting less improvement after partial meniscectomy in a study of 486 patients (*p* < 0.05). Higher baseline KOOS pain/QOL scores, higher BMI, older age, and less education were potentially modifiable risk factors that predicted less improvement after surgery (*p* < 0.05). Still, 83% (*n* = 4039) of the included patients had a successful improvement of 10 points in either KOOS Pain or KOOS-PS. In a similar vein, Krauss et al. (2021) [45] found that in a study of 509 patients, those who smoke experience postoperative improvement to a relatively similar extent as nonsmokers after partial meniscectomy. However, the overall PROM scores of smokers are lower at all time points measured. They also found KOOS ADL to be significantly different at the following timepoints: baseline for nonsmokers (61.1 ± 3.3) and smokers (53.5 ± 6.1), year 1 for nonsmokers (85.3 ± 3.1) and smokers (74.8 ± 5.2), and year 2 for nonsmokers (86.3 ± 3.2) and smokers (76.1 ± 5.7) (*p* = 0.0152, *p* < 0.00001, and *p* = 0.0005, respectively).

### 3.6. The Impact of Smoking after Meniscus Allograft Transplant

Two studies investigated the association between meniscus pathology outcomes and smoking for patients undergoing meniscus allograft transplant (MAT) with one study (50%) finding a significant association as compared to one study (50%) that did not. Waterman et al. (2016) [16] found that tobacco use was significantly associated with an increased risk of meniscus repair failure in a study of 227 patients (OR: 2.22, 95% CI, 1.19–4.17, *p* = 0.028). The group also observed a 5.7% failure rate postoperatively in the entire cohort. In contrast, Jimenez-Garrido et al. (2021) [37] found no observable difference of smoking on meniscal allograft transplantation in a study of 35 patients undergoing MAT, although the sample size was significantly smaller (*p* > 0.05). In this study, patients with a BMI ≥ 30 underwent medial meniscal transplants (88.9 vs. 42.3%, *p* = 0.022, respectively) more frequently. Following this, obese patients had higher rates of MAT failure compared with nonobese patients (adjusted hazard ratio: 11.8 [95% confidence interval: 1.5–91.4]).

### 3.7. The Impact of Smoking Postoperatively in Patients with and without Surgical Intervention

A study by Kontio et al. (2017) [38] investigated 4713 nonsurgical candidates who experienced meniscal lesions and found that smoking was not significantly associated with an increased chance of meniscal tear in former or current smokers (former smoker: (HR: 0.99, CI: 0.70–1.41), current smoker (HR: 0.88, CI: 0.62–1.25)). Bessette et al. (2019) [44] evaluate the relationship between smoking and PROMs at the time of knee arthroscopy in 638 patients with meniscal tears. They found smoking status positively associated with KOOS scores (*p* = 0.003).

### 3.8. Examination of the High-Level Evidence on Meniscus Pathology and Smoking

A total of 198 RCTs found on PubMed via a literature search were identified on secondary search with 53 RCTs meeting inclusion criteria (Supplemental Figure S1) [46,47,48,49,50,51,52,53,54,55,56,57,58,59,60,61,62,63,64,65,66,67,68,69,70,71,72,73,74,75,76,77,78,79,80,81,82,83,84,85,86,87,88,89,90,91,92,93,94,95,96,97,98]. Of these, only two (3.8%) of the RCTs examined smoking outcomes as a patient demographic variable. Hartwell et al. (2020) [65] provided information regarding smoking status (former/current) and number of patients in this category (20 smokers out of a total 96 patients). Kise et al. (2019) [75] provided only the number of patients who were smokers, eight smokers of 107 patients). From the 53 RCTs, 36 were RCTs that examined surgical outcomes and 17 were RCTs that examined nonsurgical outcomes. The two RCTs that examined smoking were surgical and nonsurgical, respectively.

## 4. Discussion

This is the first systematic review examining smoking’s impact on meniscus pathology treatment outcomes. From the 18 studies, 11 studies investigated smoking and meniscus repair with 4 studies (36.36%) reporting a significant association between smoking and poor outcomes regarding meniscus pathology. Furthermore, meta-analysis performed via six studies revealed no statistical association between smoking and failure after meniscus repair (*p* = 0.118), although this was limited by sample size. From individual articles, Pelletier et al. (2023) identified smoking as a significant risk factor for failure after medial meniscus suture repair, with an odds ratio (OR) of 5.76 (95% CI: 1.81–18.35, *p* = 0.003, *n* = 367) [13]. Similarly, Blackwell et al. (2016) noted a 3.8-fold increase in meniscus repair failure among smokers (*p* = 0.0076, *n* = 52) [14]. The higher failure rates in smokers could be reflective of smoking’s deleterious impact on tissue healing and repair mechanisms, as suggested by the existing literature on smoking’s effects on wound healing [20,21,22,23].

The current literature estimates isolated meniscus injuries to comprise 17–22% of all meniscal injuries; the remainder of meniscal injury types tend to be secondary to ACL injury [99,100,101]. When comparing isolated meniscus tears to secondary to ligamentous injury in the literature, it appears that secondary meniscus tear and smoking were associated with worse outcomes across all KOOS subscales [102]. Functionally speaking, smoking, a strong predictor for failure in ACL graft reconstructions, was shown to be the strongest predictor of postoperative meniscus tears [103]. However, we see contradicting findings in studies performed by Kontio et al., where they found no association between smoking and hospitalization due to meniscal lesions in 228 patients with meniscus tears [38]. It is essential to study the link between smoking and isolated injuries to understand its effects on meniscus repair procedures and postoperative outcomes, both functional and qualitative.

Meniscectomy is a common procedure for treating smaller, irregular, or irreparable tears of the damaged meniscus, which is a vital component for joint stability and shock absorption [1,2,3,4,104]. Successful meniscectomy entails symptom relief, restored knee function, and preventing further complications like osteoarthritis [105,106,107]. Recent studies (Jones et al., 2020, Krauss et al., 2021 [35,45]) explore smoking’s impact on meniscectomy outcomes. Jones et al. (2020) [35] proposed that current smoking was significantly associated with reduced KOOS improvement post-meniscectomy, indicating potential diminished pain relief and quality of life enhancement for smokers. Krauss et al. (2021) supported this by comparing postoperative improvements between smokers and nonsmokers [45]. Their results showed that nonsmokers consistently had higher scores than smokers at different intervals (baseline, year 1, and year 2) post-meniscectomy. This trend can also be seen in hip and knee replacement patients. Halawi et al., who studied 713 hip and knee replacement patients, found that smoking negatively affected baseline Patient-Reported Outcome Measures (PROMs), affirming Krauss et al. and Jones et. al.’s findings [35,45,108]. Such findings were paralleled by Bessette et al. (2021), who showed smoking’s significant impact on preoperative KOOS and VR-12 scores in 638 knee arthroscopy patients [44]. These studies cumulatively pose that smoking can impair long-term functional recovery and quality of life after meniscectomy, despite similar initial postoperative improvements in smokers and nonsmokers.

Smoking’s impact on meniscus transplants is a crucial topic in orthopedic research, given the procedure’s importance in treating meniscal injuries. Waterman et al. (2016) identified smoking as a significant risk factor for increased failure rates in meniscus transplant surgeries, suggesting that tobacco use may negatively affect healing and long-term success [16]. The current literature has well established that smoking overall significantly impairs tissue healing, a crucial factor in orthopedic conditions such as meniscus tears [15,17,27,109]. Smoking has been shown to decrease vascular endothelial growth factor expression and microvessel density in healing tissues, which are vital for effective wound healing [109]. In the broader scheme of orthopedic-related injuries, smoking significantly impacts the outcomes of knee ligament surgeries, such as Anterior Cruciate Ligament (ACL) and meniscus reconstruction. Current studies indicate that smokers have a higher risk of graft rupture following ACL reconstruction [110]. In the broader scope of orthopedic specialties, smoking has been linked to poorer outcomes following both knee and shoulder arthroscopy [111]. Smokers also face increased risks of soft-tissue complications in primary elective Total Knee Arthroplasty (TKA), suggesting a greater impact on knee surgeries [112]. Temporal effects are also observed; smokers face elevated rates of short-term complications undergoing procedures such as primary unicompartmental knee arthroplasty [113]. Kanneganti et al. highlight the detrimental effects of smoking on ACL reconstruction and meniscal surgery outcomes in their 2012 review paper, emphasizing the need for smoking cessation interventions in these patients [29].

Given the known impact of smoking on wound healing and injury [17,19,20,21,23,45], the limited attention to smoking in high-impact RCTs for meniscus surgery published in top orthopedic journals raises concerns. Our study adds another layer by conducting a comprehensive literature review of 198 randomized controlled trials from the top 10 orthopedic surgery journals, with 53 meeting our inclusion criteria [46,47,48,49,50,51,52,53,54,55,56,57,58,59,60,61,62,63,64,65,66,67,68,69,70,71,72,73,74,75,76,77,78,79,80,81,82,83,84,85,86,87,88,89,90,91,92,93,94,95,96,97,98]. Surprisingly, only two of these RCTs reported smoking status as a demographic factor [65,75]. This omission in the majority of RCTs (51 out of 53) represents a significant gap in orthopedic research. The lack of crucial data limits understanding of study applicability and affects surgeons’ decision-making. Without smoking status, comprehending postoperative outcomes between smokers and nonsmokers is significantly constrained. This reporting gap is troubling, given the emphasis on smoking as a key demographic factor by major medical journals due to its significant health impacts. The significant associations found in some studies suggest that preoperative counseling and treatment planning, especially for meniscus repair, should consider smoking cessation [46,47,48,49,50,51,52,53,54,55,56,57,58,59,60,61,62,63,64,65,66,67,68,69,70,71,72,73,74,75,76,77,78,79,80,81,82,83,84,85,86,87,88,89,90,91,92,93,94,95,96,97,98].

This systematic review faces limitations that necessitate a cautious approach to interpreting its outcomes. In addressing the heterogeneity observed among the included studies in our meta-analysis, several factors such as variations in study design, patient demographics, and interventions need careful consideration due to their impact on the generalizability of the findings. The mixed methodologies ranging from prospective to retrospective designs contribute differing levels of evidence, with prospective studies generally offering more robust control over confounders. This variability can lead to a wide range of effect sizes and introduce biases, affecting the overall meta-analysis results. Furthermore, the diversity in patient demographics, including age, health status, and smoking intensity, potentially affects the outcomes of meniscus repair interventions. Such disparities can alter healing rates and intervention success, thus impacting the pooled outcomes of the studies. Additionally, the variation in surgical techniques—from meniscus repairs to meniscectomies and allograft transplants—further contributes to heterogeneity. Each technique has distinct indications, recovery trajectories, and success metrics, which can skew comparative analyses. We employed a random-effects model in our meta-analysis, which assumes a distribution of effects across studies, providing a more generalized conclusion that accounts for variations beyond chance. The I^2^ statistic was used to quantify heterogeneity, indicating a substantial diversity that necessitates cautious interpretation of the pooled estimates. The study quality further influences the overall conclusions of our meta-analysis. Studies were evaluated using the MINORS scale, resulting in a mix of moderate and low-quality ratings. Including studies across a spectrum of quality levels introduces potential biases and affects the robustness of the findings. To mitigate this, future analyses will incorporate sensitivity analyses that exclude lower-quality studies to assess their impact on the overall results. This approach will enhance the precision of our effect estimates and strengthen the reliability of our conclusions, providing more definitive guidance for clinical practice.

A notable gap in our review is the absence of RCTs directly comparing smokers and nonsmokers in knee ligament or meniscus surgeries, limiting our capacity to establish clear causal links. Our secondary search further highlighted the lack of smoking status as a demographic variable in high-level orthopedic research, and the implications of this are profound. The power of RCTs comes from the ability to provide crucial information regarding variables of interest while accounting for variation in patient characteristics and patient behavior. Smoking, a very common lifestyle habit of many patients, is crucial to account for in these controlled studies given the implications the literature has shown on postoperative outcomes and healing. Enhancing future research to account for these variables is crucial, particularly in areas where smoking markedly influences surgical outcomes. The call for more rigorous and standardized research is called for to overcome the current limitations to better understand the role of smoking in surgical recovery and outcomes.

## 5. Conclusions

Mixed evidence exists regarding smoking’s impact on meniscus pathology outcomes, with many low- to moderate-quality observational studies and limited high-quality data. Although smoking may potentially influence success rates in meniscal repair and allograft transplant, this association lacks consistent confirmation and warrants further investigation. Meta-analysis from a subset of articles found no significant link between smoking and meniscus repair failure, but the limited sample size constrains this conclusion. Smoking did not show adverse outcomes after meniscectomy, but there is a scarcity of relevant articles. Furthermore, of all the RCTs related to meniscus in the top orthopedic journals, only a mere 3.8% reported patient smoking status, indicating a lack of research in this area. Insufficient data also exist on the impact of pack-years smoked on meniscal pathology and patient outcomes. To provide more definitive conclusions and better guide preoperative counseling and planning, more comprehensive and controlled studies are necessary. We strongly recommend and emphasize the importance of including smoking status as a standard demographic variable in such future studies.

## Figures and Tables

**Table 1:** The top 10 journals searched for randomized controlled trials, and the associated search terms for each respective journal.

Journal Name	Search Terms
*The American Journal of Sports Medicine*	“Am J Sports Med” [Journal]
*Journal of Bone and Joint Surgery*	“J Bone Joint Surg Am” [Journal]
*Journal of Orthopedic & Sports Physical Therapy*	“J Orthop Sports Phys Ther” [Journal]
*Knee Surgery, Sports Traumatology, and Arthroscopy*	“Knee Surgery, Sports Traumatology, Arthroscopy: Official Journal of the ESSKA” [Journal]
*Bone and Joint Journal*	“Bone Joint J” [Journal]
*Osteoarthritis and Cartilage*	“Osteoarthritis Cartilage” [Journal]
*Clinical Orthopedics and Related Research*	“Clinical Orthopaedics and Related Research” [Journal]
*Acta Orthopedica*	“Acta Orthopaedica” [Journal]
*The Journal of Arthroplasty*	“The Journal of Arthroplasty” [Journal]
* The Journal of Physical Therapy *	“J Physiother” [Journal]

**Table 2:** The Methodological Index for Non-Randomized Studies (MINORS) grading for all included articles in this systematic review.

First Author (Year)	Study Type	Total Score	Cl-Ear Aim	Consecutive Patients Included	Prospective Data Collection	Appropriate Endpoints	Unbiased Study Endpoint Assessment	Appropriate Follow-Up	Less Than 5% Lost to Follow-Up	Prospective Study Size Calculation	Adequate Control	Contemporary Group	Baseline Equivalence	Adequate Statistical Analysis
Gupta et al. (2023) [32]	Non-comparative	8	2	2	0	2	0	2	0	0	-	-	-	-
Clinic et al. (2020) [42]	Comparative	14	2	2	2	2	0	2	0	0	0	2	0	2
Blackwell et al. (2016) [14]	Comparative	18	2	2	0	2	0	2	0	2	2	2	2	2
Zabrzyński et al. (2022) [41]	Comparative	18	2	2	0	2	2	2	2	0	2	2	0	2
Buyukkuscu et al. (2019) [34]	Non-comparative	10	2	2	0	2	0	2	2	0	-	-	-	-
Laurendon et al. (2017) [39]	Non-comparative	10	2	2	0	2	0	2	2	0	-	-	-	-
Santana et al. (2022) [40]	Comparative	14	2	2	0	2	0	2	0	0	2	2	0	2
Haklar et al. (2013) [36]	Comparative	14	2	2	0	2	0	2	0	2	0	2	0	2
Waterman et al. (2016) [16]	Non-comparative	10	2	2	0	2	0	2	2	0	-	-	-	-
Pelletier et al. (2023) [13]	Comparative	12	2	2	0	2	0	2	0	0	0	2	0	2
Kontio et al. (2017) [38]	Non-comparative	10	2	2	0	2	0	2	2	0	-	-	-	-
Saltzman et al. (2020) [33]	Comparative	14	2	2	0	2	0	2	0	0	2	2	0	2
Jones et al. (2020) [35]	Non-comparative	10	2	2	2	2	0	2	0	0	-	-	-	-
Domzalski et al. (2021) [15]	Comparative	16	2	2	0	2	0	2	0	2	2	2	0	2
Jiménez-Garrido et al. (2021) [37]	Comparative	12	2	2	0	2	0	2	0	0	0	2	0	2
Kraus et al. (2021) [45]	Comparative	16	2	2	0	2	0	2	2	0	2	2	0	2
Bessette et al. (2019) [44]	Non-comparative	12	2	2	2	2	0	2	2	0	-	-	-	-
Uzun et al. (2019) [43]	Comparative	18	2	2	0	2	0	2	2	0	2	2	2	2

**Table 3:** Outcomes study table with important findings and demographics.

First Author (Year)	Number of Patients (*n*)	Sex (Number Male/Female)	Average Age (Years)	Average Follow-Up (Months)	Main Findings
Gupta (2023) [32]	205	Male (*n* = 157) Female (*n* = 48)	24.42	12.52	Smoking has no effect on failure of meniscal repairs.
Saltzman (2020) [33]	75	Male (*n* = 47) Female (*n* = 28)	26.53	23.41	Smoking not significantly associated with failure.
Buyukkuscu (2019) [34]	33	Male (*n* = 23) Female (*n* = 10)	46.1	31.1	Smoking was not significantly associated with clinical and functional improvement after meniscal repair.
Santana (2022) [40]	432	Male (*n* = 174) Female (*n* = 258)	62.2	72	There was no significant difference in smoking status between patients who had no tear and patients who had a tear with or without surgical intervention.
Pelletier (2023) [13]	367	Male (*n* = 268) Female (*n* = 99)	28	98	Smoking was significantly associated with bucket-handle tears.
Jimenez-Garrido (2021) [37]	35	Male (*n* = 32) Female (*n* = 3)	36.6	75.7	No observable difference of smoking on meniscal allograft transplantation.
Jones (2020) [35]	486	Male (*n* = 265) Female (*n* = 221)	55	12	Current smoking predicted less improvement for all outcomes except KOOS-PS after arthroscopic partial meniscectomy.
Laurendon (2017) [39]	87	Male (*n* = 61) Female (*n* = 26)	28.3	31	Smoking was not significantly associated with failure.
Haklar (2013) [36]	112	Male (*n* = 94) Female (*n* = 18)	34.57	48.39	Smoking had no effect on healing in isolated meniscal tears.
Kontio (2017) [38]	4713	Male (*n* = 2320) Female (*n* = 2393)	57.35	276	Smoking not significantly associated with tears in current and former smokers.
Zabrzynski (2022) [41]	50	Male (*n* = 32) Female (*n* = 18)	41.68	6	Smoking was not significantly associated with functional repair outcomes.
Blackwell (2016) [14]	52	Male (*n* = 32) Female (*n* = 20)	27.9	13	Meniscus repair failure was 3.8 times higher for smokers vs. nonsmokers.
Domzalski (2021) [15]	92	Male (*n* = 36) Female (*n* = 56)	31.5	37.4	Function was significantly better in nonsmokers compared to smokers.
Waterman (2016) [16]	227	Male (*n* = 203) Female (*n* = 24)	27.2	25.68	Tobacco use was significantly associated with increased risk of failure.
Clinic (2020) [42]	145	Male (*n* = 102) Female (*n* = 43)	18	12	Smoking had no effect on healing in isolated meniscal tears.
Kraus (2021) [45]	509	Male (*n* = 206) Female (*n* = 301)	47.58	24	Smokers will improve a relatively similar amount as nonsmokers after partial meniscectomy, but their overall PROM scores are lower.
Bessette (2019) [44]	638	Male (*n* = 332) Female (*n* = 306)	55.1	-	Smoking positively associated with Knee Injury and Osteoarthritis Outcome Score.
Uzun (2019) [43]	43	Male (*n* = 37) Female (*n* = 6)	29.5	63.2	Smoking was identified as a risk factor for repair failure.

**Table 4:** Failure rate after meniscus repair stratified by smoking status.

First Author (Year)	Group (Smoker/Nonsmoker)	Total Pts (in Each Group)	# of Postop Failures (*n*, %)
Gupta (2023) [32]	Smoker	24	4, (16.7%)
	Nonsmoker	181	35, (19.3%)
Saltzman (2020) [33]	Smoker	4	0, (0%)
	Nonsmoker	74	15, (20.3%)
Haklar (2013) [36]	Smoker	9	2, (22.2%)
	Nonsmoker	14	3, (21.4%)
Blackwell (2016) [14]	Smoker	52	15, (28.84%)
	Nonsmoker	52	4, (7.69%)
Domzalski (2021) [15]	Smoker	47	5, (10.63%)
	Nonsmoker	45	3, (6.67%)
Uzun (2019) [43]	Smoker	5	3, (60%)
	Nonsmoker	21	2, (9.52%)

## Data Availability

The data presented in this study are available from the corresponding author.

## Supplementary Materials

The following supporting information can be downloaded at: https://www.mdpi.com/article/10.3390/life14050584/s1, Figure S1: Preferred Reporting Items for Systematic Reviews and Meta-Analyses (PRISMA) diagram for the secondary search of this study.
