# Supplementary material for: Examining the Evidence Regarding Smoking and Patient Outcomes for Isolated Meniscus Pathology: A Comprehensive Systematic Review and Meta-Analysis
Source: Life (Basel). 2024 Apr 30;14(5):584. doi: 10.3390/life14050584 (PMC11122235; doi:10.3390/life14050584)
Supplement: Supplementary file 1 [file life-14-00584-s001.zip › life-2910096-supplementary.pdf]

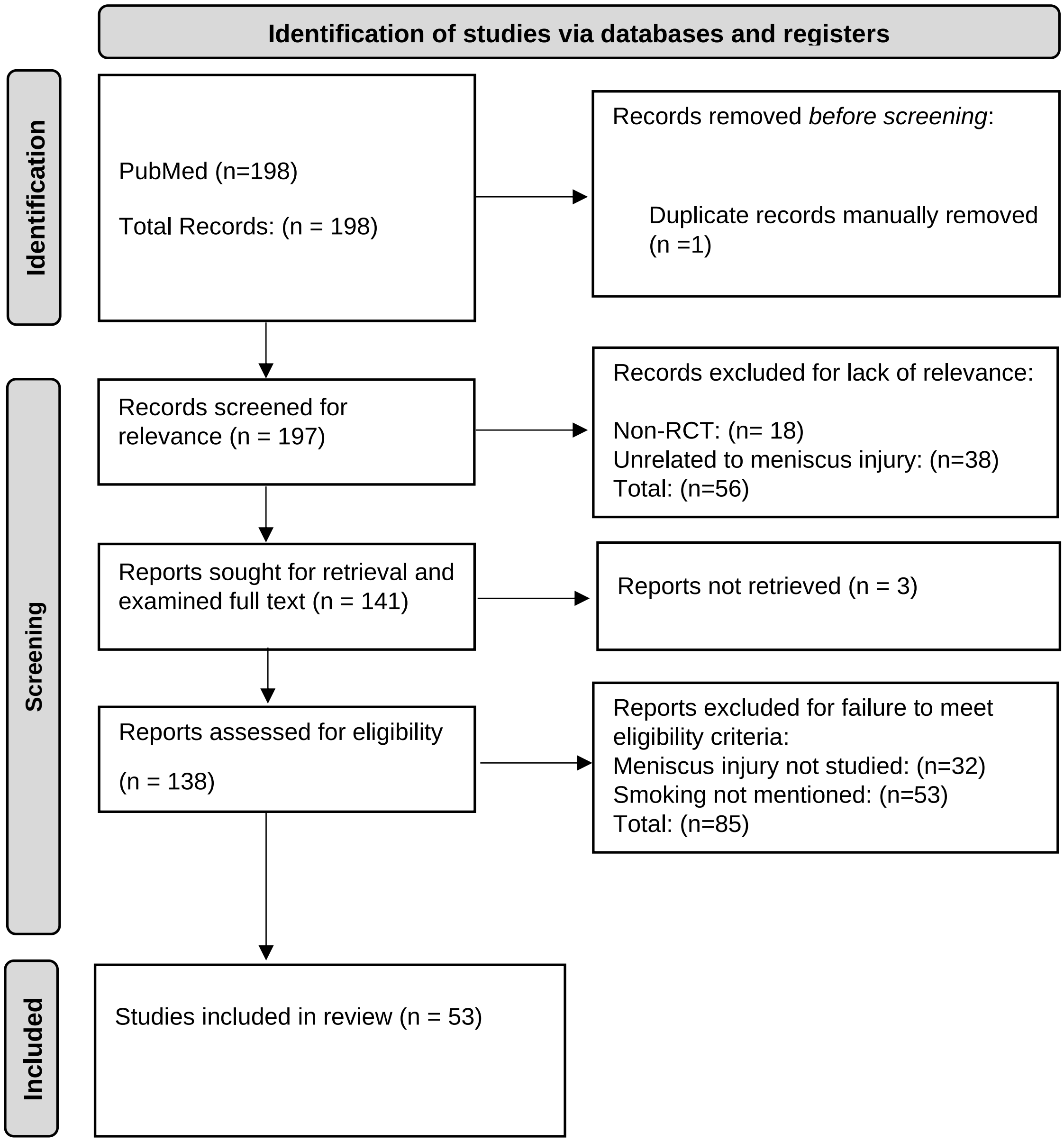


**Supplemental Figure S1**: Preferred Reporting Items for Systematic Reviews and Meta-Analyses (PRISMA) diagram for the secondary search of this study.
